# Integrated metabolomics and lipidomics evaluate the alterations of flavor precursors in chicken breast muscle with white striping symptom

**DOI:** 10.3389/fphys.2022.1079667

**Published:** 2023-01-18

**Authors:** Fuli Kong, Lu Bai, Zhengxiao He, Jiahong Sun, Xiaodong Tan, Di Zhao, Furong Feng, Dawei Liu, Guiping Zhao, Jie Wen, Ranran Liu

**Affiliations:** ^1^ Institute of Animal Sciences, Chinese Academy of Agricultural Sciences, Beijing, China; ^2^ State Key Laboratory of Animal Nutrition, Key Laboratory of Animal (Poultry), Genetics Breeding and Reproduction, Ministry of Agriculture, Beijing, China; ^3^ Foshan Gaoming Xinguang Agricultural and Animal Industrials Corporation, Foshan, China

**Keywords:** broiler chickens, white striping, lipidomics, metabolomics, flavor precursors

## Abstract

White striping (WS) is the most common myopathy in the broiler chicken industry. To reveal flavor changes of WS meat objectively, flavor precursors of WS breast muscle were evaluated systematically with integrated metabolomics and lipidomics. The results showed that WS could be distinguished from normal controls by E-nose, and four volatile compounds (o-xylene, benzene, 1,3-dimethyl, 2-heptanone and 6-methyl and Acetic acid and ethyl ester) were detected as decreased compounds by gas chromatography-mass spectrometry. Lipidomic analysis showed that WS breast fillets featured increased neutral lipid (83.8%) and decreased phospholipid molecules (33.2%). Targeted metabolomic analysis indicated that 16 hydrophilic metabolites were altered. Thereinto, some water-soluble flavor precursors, such as adenosine monophosphate, GDP-fucose and L-arginine increased significantly, but fructose 1,6-bisphosphate and L-histidine significantly decreased in the WS group. These results provided a systematic evaluation of the flavor precursors profile in the WS meat of broiler chickens.

## 1 Introduction

Chicken, especially the breast muscle, is widely regard as an important source of high quality protein with low lipid content (Marangoni et al., 2015). Modern broilers have been intensively selected for an accelerated growth rate and increased breast yield. Consequently, growth-related breast muscle abnormalities have been reported successively in recent years. Studies have shown that factors influencing the incidence of the WS symptom are breast weight, dietary nutrition, dietary management, inheritance, and gender (Kuttappan et al., 2012a; Kuttappan et al., 2013; Trocino et al., 2015; Malila et al., 2018). WS is featured with white striations parallel to muscle fibers (Kuttappan et al., 2012a). WS incidence has varied from 12% up to 98% in broilers (Kuttappan et al., 2012a; Petracci et al., 2013; Tijare et al., 2016; Kuttappan et al., 2017; Malila et al., 2018; Golzar Adabi and Demirok Soncu, 2019). According to the degree of WS partitioning, the affected breasts were divided into normal, moderate, severe, or extreme (Kuttappan et al., 2016). De carvalho et al. (2020) showed that the occurrence of moderate WS (9.6%–92.5%) was always well above severe WS (0.0%–2.5%), which was consistent with a report by Golzar Adabi and Demirok Soncu, (2019). Breast fillets with severe scores are not suitable for fresh retails due to an unacceptable appearance, but breasts with a moderate score are common in the supermarket. Thus, moderate WS deserves further study in the respect of flavor quality.

In general, the disadvantages of WS meat quality include the appearance, nutritional value and processing characteristics (water holding capacity) (Kuttappan et al., 2012b; Petracci et al., 2013; Baldi et al., 2018). Moderate WS does not have a significant impact on cook loss, marinade uptake and Meullenet-Owens razor shear energy (Tijare et al., 2016) and texture attributes (such as juiciness, rate of breakdown, chewiness, cohesiveness and hardness) (Sanchez Brambila et al., 2016). Therefore, moderate WS with minor changes in appearance has few effects on meat quality.

It is well-known that intramuscular fat (IMF), composed of triacylglycerols (TGs), phospholipids, etc., is an important factor to increase meat quality (Cui et al., 2012; Jiang et al., 2017; Qiu et al., 2017). A certain amount of IMF content can enhance flavor, tenderness, water retention and the sensory quality of meat (Suzuki et al., 2005; Zhao et al., 2007). It was reported that phospholipids contribute significantly to pork and beef flavor (Mottram 1998; Huang et al., 2010). Gandemer (2002) although TGs were also important for flavor formation as TGs contributed 30%–50% of free fatty acids. WS was associated with a greater amount of lipids and moderate scored WS fillets were also rich in polyunsaturated fatty acids (Kuttappan et al., 2012a; Golzar Adabi and Demirok Soncu, 2019). However, there are limited systematic evaluations of flavor precursors in WS meat. In this study, integrated lipidomics and metabolomic analyses were applied to reveal volatile compounds, lipids and hydrophilic metabolite changes in WS meat.

## 2 Materials and methods

### 2.1 Broiler husbandry and sample collection

One hundred male Arbor Acres (AA) commercial broilers were raised in the Institute of Animal Sciences at the Chinese Academy of Agricultural Sciences (IASCAAS, Beijing, China) experimental base. Broilers were fasted for 12 h and weighed individually at 42 d of age. All the chickens were electrically stunned and slaughtered. The samples about 20 g of the right breast muscle from the cranial part were collected and stored at −80°C. The samples were crushed and mixed after defrosted at 4°C, two portions of 6 g samples were weighed and used for E-nose and GC/MS test separately. The left part was deboned and stored for 3 h at 4°C before scoring. Five moderate WS breast fillets and five normal samples selected were used for the E-nose and volatile compounds measurement.

A total of 150 male fast-growing white feathered pure line B were raised by Xinguang Agricultural and Animal Industrial Co., Ltd. (Mile, China). The chickens were fasted for 12 h and weighed individually at 42 d of age. The samples from the superficial layer of the cranial part of the right breast muscle were collected and stored at −80°C for next step analyses. Other procedures such as selection and slaughter of chickens, deboned left breast and WS scoring were the same as in the AA broilers flock. Five moderate WS breast fillets and five normal samples selected were used for omics analysis.

All chickens were raised individually in three-story step cages and the environmental conditions and nutritional compositions were same as recommended by the Feeding Standard of Chickens in China (NY 33-2004). The diets and water were available *ad libitum*. The guidelines established by the Ministry of Science and Technology (Beijing, China) was the reference standard of this experimental animals. All procedures were approved by the ethics committee of the Institute of Animal Sciences in the CAAS and the reference number was IAS 2019-44.

### 2.2 Phenotype identification and sample selection

The left breast fillets of 100 AA commercial broilers and 150 pure line B chickens were scored. Briefly, the score included normal breast score = 0, moderate WS score = 1 (narrow fat white lines, <1 mm wide), severe WS score = 2 (fat white lines 1–2 mm wide and visible on the surface) and extreme WS score = 3 (white bands >2 mm wide and covering the whole surface) (Kuttappan et al., 2016). The compression test was introduced to identify fillet with WB.The compression force of fillets was measured as described by Sun et al. (2018). Fillets were measured on cranial regions and compressed to 20% with a 6-mm flat probe on a TA.XT Plus Texture Analyzer (Stable Micro Systems Ltd., Godalming, United Kingdom). The parameters were set as follows: trigger force was 5 g, probe height was 55 mm, pre- and post-probe speeds were 10 mm/s and the test probe speed was 5 mm/s. Each fillet was tested three times at different regions of the fillet.

The breast tissues of pure line B were used to estimate the triglyceride (TG) content commercial kits (Nanjing Jiancheng Bioengineering Institute, Nanjing, China). In brief, procedures were as follows: add 18 ml absolute ethyl alcohol solution in the tube with 2 g breast tissue and ground for 2 min with a tissue grinder, centrifuged at 2,500 rmp for 10 min at 4°C, then obtain the supernatant for TG determined.

### 2.3 Electronic nose analysis

The five moderate WS and five control samples from AA chickens were defrosted at 4°C, and 6.0 g of each sample were placed in sealed sample bottles, and the ratio of water to meat is 2:1. Subsequently, the bottles were boiled at 100°C for a further 30 min (Jin et al., 2021). The E-nose used in the experiment was a Heracles II moderate (Alpha MOS, Toulouse, France) based on fast gas chromatography (GC). There are two ultrasensitive flame ionization detectors (μ-FIDs) and two capillary columns of different polarity including a non-polar MTX-5 (5% diphenyl) and a medium polarity MXT1701 (14% cyanopropyl-phenyl) dimensioning as 10 m × 0.18 mm × 0.4 μm. There are also an Odor Scanner HS 100 autosampler (Gerstel GmbH, Mülheim, Germany) equipped with the E-nose. All samples were incubated at 50°C for 30 min and agitation at 500 rpm. The headspace phase (3,000 μL) was migrated to the injector and heated to 200°C. The parameters were as follows: initial temperature at 70°C for 29 s, then ramped at 2°C/s to 250°C for 34 s, and the detector temperature was at 270°C. The carrier gas was Hydrogen N5.0 (Linde Gaz, Krakow, Poland).

### 2.4 Analysis of volatile compounds

The same pre-treated were applied as those for the electronic nose analysis. The solid-phase microextraction (SPME) arrows were directly migrated and desorbed in the injection port of the GC for 3 min on splitless mode after headspace extraction. GC-mass spectrometry (MS) (Thermo Fisher Scientific, United States) with a TG-WAX capillary column (30 m × 0.25 mm, 0.25 μm film wide, Thermo Fisher Scientific, Waltham, MA, United States) were used for volatile compounds analysis. Firstly, the column temperature was kept at 40°C for 2 min, then slowly increased to 230°C at a rate of 4°C/min and finally kept at 230°C for 5 min. The mass spectrometer parameters were as follows: ion source temperature 280°C, interface temperature 250°C and electron ionization (EI) 70 eV; MS detection was full scan mode (mass range of 30-400 m/z). Each chromatographic peak corresponded by each spectrogram was qualitatively determined according to the computer chart. The mass spectra were compared with information from the National Institute of Standards and Technology and Wiley libraries and the linear retention indices (LRIs) was matched with the online database to confirmed all the information (http://www.flavornet.org/https://pubchem.ncbi.nlm.nih.gov/), then the volatile compounds were identified.

### 2.5 Lipidomics analysis

Five controls and five moderate WS samples were selected in pure line B. The lipids were extracted as follows: added 650 μL chloroform/methanol (2:1 v/v) into 65 mg sample and homogenized by a superfine homogenizer. The 150 μL water was added; samples were vortexed for 2 min and incubated for 10 min at 4°C, then centrifuged at 3,000 rpm for 15 min at 4°C. Transferred the lower chloroform layer into a new tube (1.5 ml, Eppendorf, Wesseling-Berzdorf, Germany) and aqueous washing was repeated twice again. The extracts were dried in a Thermo Scientific Savant Vac (ThermoFisher, Waltham, MA) for 1.5 h and dry pellets were stored at -80°C. The pellet was dissolved and protein concentration was determined and normalized to the protein level for the later detection.

Untargeted lipidomics test was carried out with the Q-Exactive Orbitrap mass spectrometer coupled to a UPLC system Ultimate 3,000 (Thermo Fisher). The UPLC system was coupled to a Q-Exactive HFX Orbitrap mass spectrometer (Thermo Fisher) which was furnished with a heated electrospray ionization (HESI) probe. Lipid extracts were obtained by a Cortecs C18 column (100 × 2.1 mm, Waters, Milford, MA, United States). A binary solvent system was utilized. The mobile phase A consisted of Acetonitrile (ACN): H2O (60:40), 10 mM ammonium acetate, and the mobile phase B included iso-propyl alcohol (IPA): ACN (90:10), 10 mM ammonium acetate. A 35-min gradient was applied with a flow rate of 220 μL/min. The sample tray and column chamber were preserved at 10°C and 40°C, respectively.

The data were obtained through data dependent acquisition by MS/MS. The mass spectrometer parameters were set as follows: a spray voltage of 3,200 V (positive) and 2,800 V (negative), an auxiliary gas flow rate of 10 Arb units, a capillary temperature of 320°C, an NCE of 15/30/45, a mass range (m/z) of 240–2,000 (positive) and 200–2,000 (negative), and a topN of 10. A full scan and fragment spectra were acquired with a resolution of 70,000 and 17,500, respectively. Lipid identification were carried out by LipidSearch software (Version 4.1.16; Thermo Fisher). The relative quantification of the identified lipids were calculated from their relative peak areas.

### 2.6 Metabolomics analysis

Five controls and five moderate WS from pure line B were used for metabolite extraction and metabolomics analysis based on MS. Added HPLC-grade methanol (700 μL, 80% v/v) which was cooled at −80°C for 2 h to the sample (70 mg). The mixture was ground in dry ice by a superfine homogenizer, vortexed for 3 times (10 s per time) incubated at −80°C for 8 h and centrifuged at rmp 12.000 at 4°C for 20 min. The supernatant of the sample was diverted to a new tube (1.5 ml, Eppendorf) and concentrated by a Thermo Scientific Savant Vac for 4 h to dryness. 80% methanol was used for the redissolution of residues to furthur analyze.

Targeted metabolomic analysis was implemented by TSQ Quantiva (Thermo Fisher Scientific). Reverse-phase chromatography (C18 column) was used with 10 mM tributylamine, 15 mM acetate in water as mobile A and 100% methanol as mobile phases B. The emphasis of this analysis was the TCA cycle, glycolysis pathway, amino acids, purine metabolism and pentose phosphate pathway. In this experiment, we used a 25-min gradient from 5% to 90% as mobile B. Data acquisition form the positive-negative ion switching mode. The resolving power of Q1 and Q3 are both 0.7 FWHM. The source voltage positive-negative ion mode was 3,500 v and 2,500 v respectively. The source work conditions were as follows: heater temperature, 300°C; auxiliary gas flow rate, 10; sheath gas flow rate, 35; spray voltage, 3,000 v; capillary temperature, 320°C. Tracefinder 3.2 (Thermo Fisher Scientific) with a home-built database was used for the metabolite identification.

### 2.7 Statistical analysis

The SPSS 25.0 software (SPSS, Inc., Chicago, IL) was utilized. Statistical evaluation was carried out by the Student’s two-tailed t-test. Data are displayed as means ± standard error. Statistical significance was set as *p* < 0.05. Principal component analysis (PCA) and pathway analysis were performed with MetaboAnalyst 5.0. Graphs were generated with GraphPad Prism 8.0 software (GraphPad Software Inc., La Jolla, CA).

## 3 Results

### 3.1 Phenotype identification and sample selection

**FIGURE 1:**
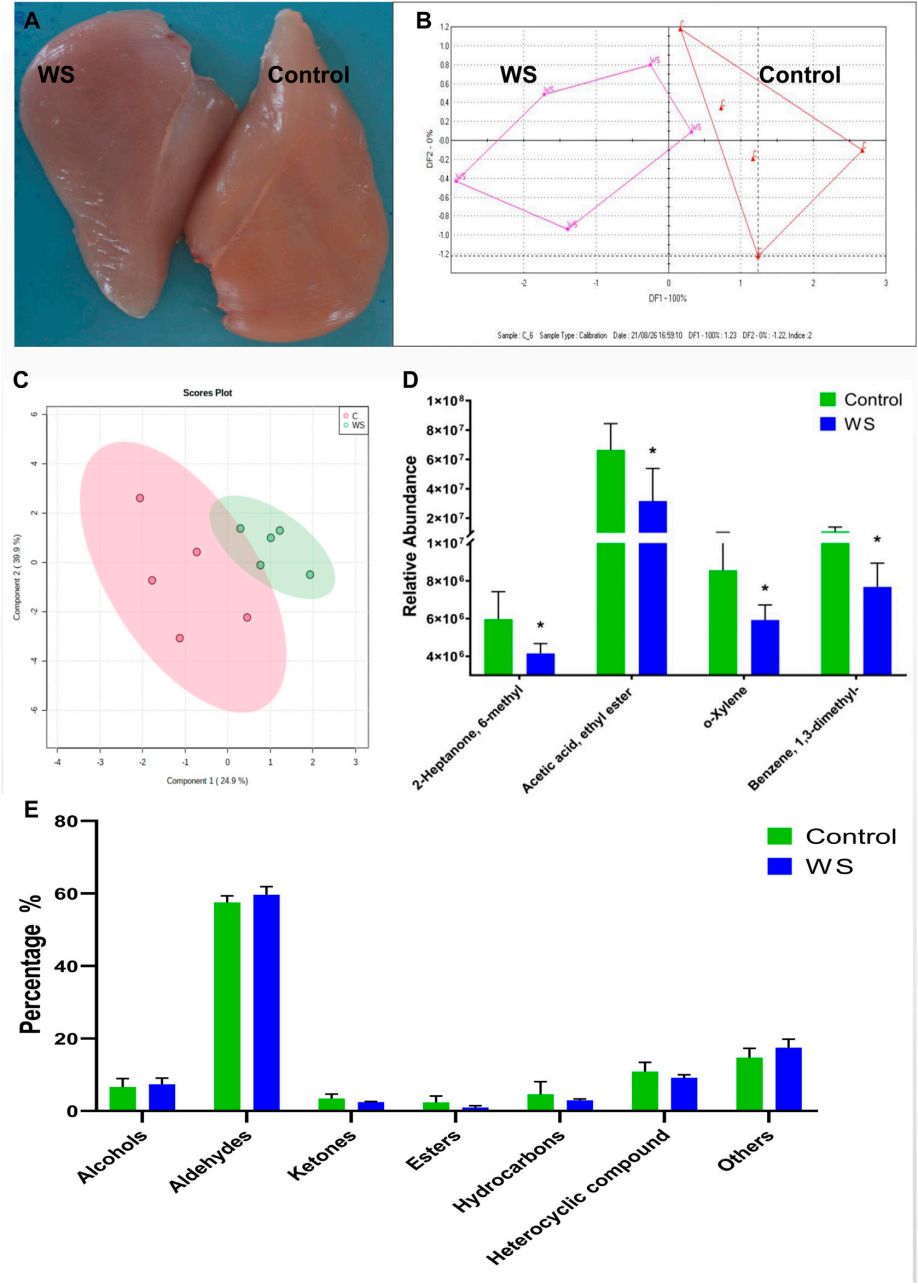
The differences between the moderate WS and control breast fillets in AA chickens. **(A)** representative WS (scores 1) and control breast muscle. **(B)** E-nose results was displayed by the discriminant factor analysis (DFA). **(C)** Volatile compounds in the WS and normal breast fillets showed by partial least squares-discriminant analysis (PLS-DA) score plots. **(D)** Relative abundance of significant differential volatile flavor molecules between the two groups. **(E)** The percentage of volatile categories identified in breast fillets (*p < 0.05).

In the 100 AA chickens measured, there were eight (8%) moderate WS, six (6%) severe WS, 27 (27%) WB and 35 (35%) WB accompanied by WS were found. The compression force, the body weight and breast weight were similar between WS and controls (Table 1). In the 150 pure line B chickens, there were 23 (15%) moderate WS, 43 (29%) WB and 25 (17%) WB accompanied by WS were found. The compression force and TG remarkably increased in the WS group, but the average body weight and breast weight were similar. The figures of WS breast with score 1 and controls are displayed in Figure 1A. The samples with severe WS or WB were excluded in the following test.

**TABLE 1:** The compression force, breast weight and body weight between the control and moderate WS groups in AA and pure line B chickens.*

Category	Control	MOD WS	P
Commercial AA			
Chicken number	24 (24%)	8 (8%)	-
WS Score	0	1	-
Compression force/(N)	2.45 ± 0.62	3.00 ± 0.47	*p* > 0.05
Breast weight/(g)	207 ± 18	221 ± 18	*p* > 0.05
Body weight/(g)	2157± 133	2127 ± 178	*p* > 0.05
Pure line B			
Chicken number	59 (39%)	23 (15%)	-
WS Score	0	1	-
Triglyceride (TG)/(mg/kg)	5.85 ± 0.91	14.70 ± 2.09***	0.021
Compression force/(N)	2.57 ± 0.87	3.81 ± 0.41*	*p* > 0.05
Breast weight/(g)	241 ± 22	250 ± 38	*p* > 0.05
Body weight/(g)	2531 ± 151	2565 ± 137	*p* > 0.05

^*^ The numbers of samples with severe WS or WB were not supplied here.

### 3.2 Analysis of volatile organic compounds (VOCs) in WS and normal breast fillets

Moderate WS samples were selected from AA broilers and measured using E-nose. The WS meat could be distinguished with discriminant factor analysis (DFA) (Figure 1B). The changes of VOCs in WS fillets were further measured by GC-MS. Overall, 101 VOCs from breast fillets were identified (Supplementary Table S1). These VOCs were classified into seven categories: alcohols (8), aldehydes (27), ketones (16), esters (6), hydrocarbons (17), heterocyclic compounds (18) and other compounds (9). Based on the PLS-DA score plot, there was some overlap between the control and moderate WS group (Figure 1C). For hydrocarbons, the relative intensity of o-xylene and benzene, 1,3-dimethyl in WS significantly decreased (Figure 1D). Regarding ketones and esters, levels of 2-heptanone, 6-methyl and acetic acid and ethyl ester in the WS group were remarkably lower than in the controls (Figure 1D). There is no significant difference of the percentage of volatile categories between the control and moderate WS groups (Figure 1E).

### 3.3 Lipidomic analysis of WS and normal breast fillets

**FIGURE 2:**
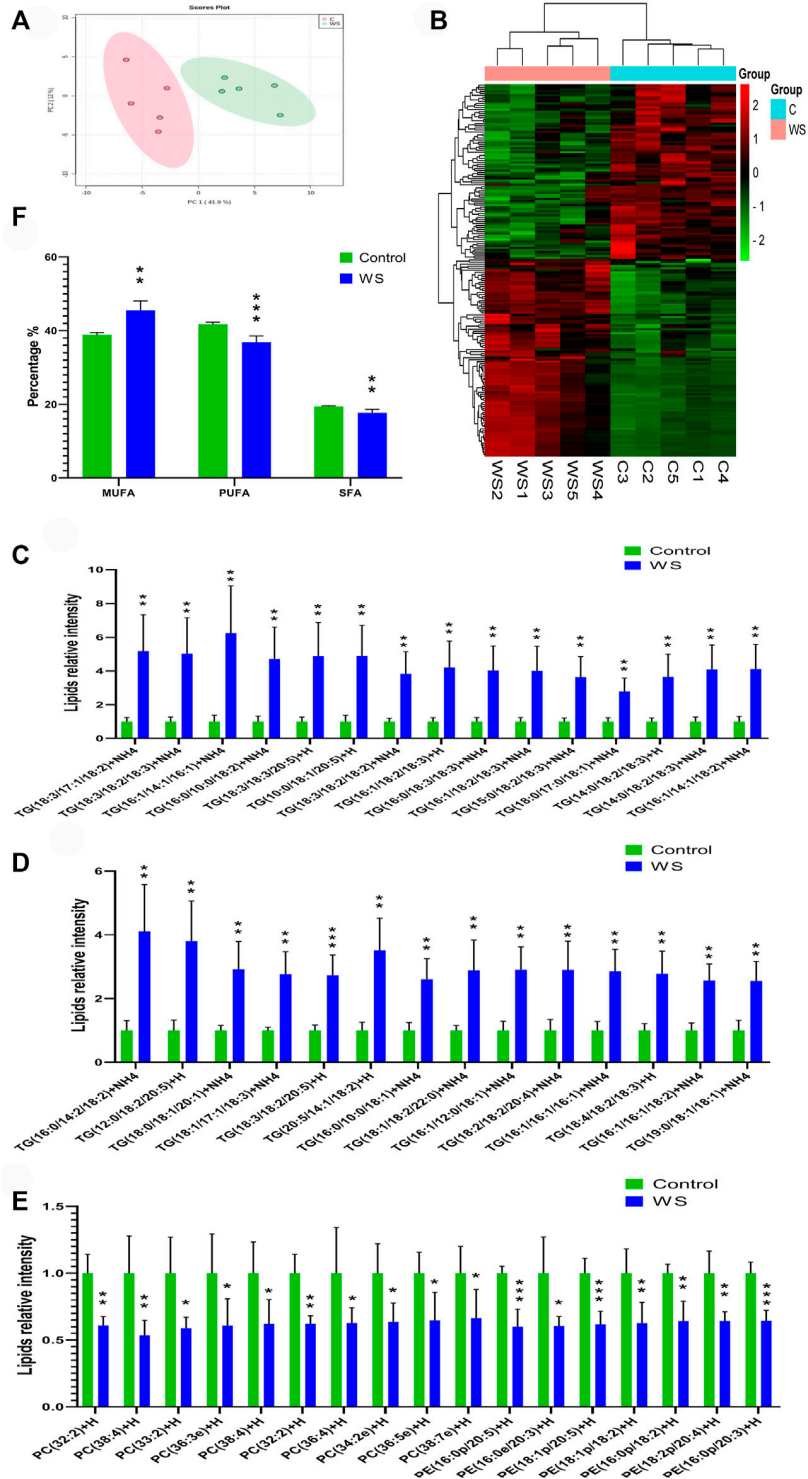
The lipid profiles of moderate WS and control breast fillets in pure line B chickens. **(A)** Principal component analysis (PCA) score plots of all the lipid molecules in the WS and control. **(B)** Heat map of significantly different lipid molecular categories influenced by WS. Increased and decreased lipids molecules were represented by red and green cells of the heat map respectively. **(C–D)** Differential abundance of TG lipid molecules in the WS group higher than 2.5-fold change of controls. **(E)** Differential abundance of PC and PE lipid molecules in the WB-affected group lower than 66.7% of the controls. **(F)** Percentages of MUFAs, PUFAs and SFAs in FAs. TGs, triglycerides; PC, phosphatidylcholine; PE, phosphatidylethanolamine; SFAs, saturated fatty acids; MUFAs, monounsaturated fatty acids; PUFAs, polyunsaturated fatty acids (*p < 0.05, **p < 0.01, ***p < 0.001).

There are 560 lipid molecules identified form the positive and negative ion mode in breast fillets of pure line B (Supplementary Table S2) including 20 lipid categories. The major lipid categories were phosphatidylcholine (PC, 138), phosphatidylethanolamine (PE, 133) and TG (108) (Table 2, Supplementary Table S3). PCA analysis demonstrated discrimination between the control and WS fillets (Figure 2A). The lipid molecules with significantly differential abundance in WS are shown in Figure 2B and Table 2. Compared with the control group, TGs increased in 90 categories whereas PCs in 52 categories and PEs in 38 categories decreased. In total, 29 categories of TGs increased 2.5-fold-6.2-fold as shown in Figures 2C,D. Changes in PC and PE lipid molecules were lower than 66.7% of the controls (Figure 2E). The ranges of 10 PC categories decreased from 33.5% to 46.4% and 7 categories of PEs changed from 35.6% to 40.0%. As shown in Figure 2F, the relative level of monounsaturated fatty acids (MUFAs) was remarkably increased in the WS (45.48%) than in the controls (38.85%). The level of SFAs and PUFA was significantly decreased in the WS (36.82%, 17.69%, respectively) than in the controls (41.77%, 19.38%, respectively).

**TABLE 2:** The number of increased and decreased lipid molecules in different lipid categories of the WS group in the pure line B chickens.

Lipid	PC	PE	TG	DG	SM	Cer	Co	FA	PG	CL	SPHP	PS	Others	Total
Total	138	133	108	3	31	27	2	31	9	40	1	3	34	560
Changed	53	41	93	3	11	3	1	1	1	1	1	1	-	210
Up	1	3	90	3	-	-	-	1	1	1	-	-	-	100
Up ratio %	0.7	2.3	83.3	100	3.2	11.1	50	3.2	11.1	2.4	-	-	-	17.9
Down	52	38	3	-	11	3	1	-	-	-	1	1	-	110
Down ratio %	37.7	28.6	2.8		35.5	11.1	50	-	-	-	100	33.3	-	19.6

PC, phosphatidylcholine; PE, phosphatidylethanolamine; TG, triglyceride; DG, diglyceride; SM, sphingomyelin; Cer, ceramides; Co, coenzyme; FA, fatty acid; PG, phosphatidylglycerol; CL, cardiolipin; SPHP, lysophosphatidylserine; PS, phosphatidylserine. Others included: CerP, ceramide phosphate; LPC, lysophosphatidylcholine; AcCa, acyl carnitine; HexlCer, simple Glc series; PA, phosphatidic acid; PS , phosphatidylserine; PI, phosphatidylinositol; LPE, lysophosphatidylethanolamine; LPS, lysophosphatidylserine.

### 3.4 Hydrophilic metabolites changed by WS symptom in breast according to targeted metabolomics

**FIGURE 3:**
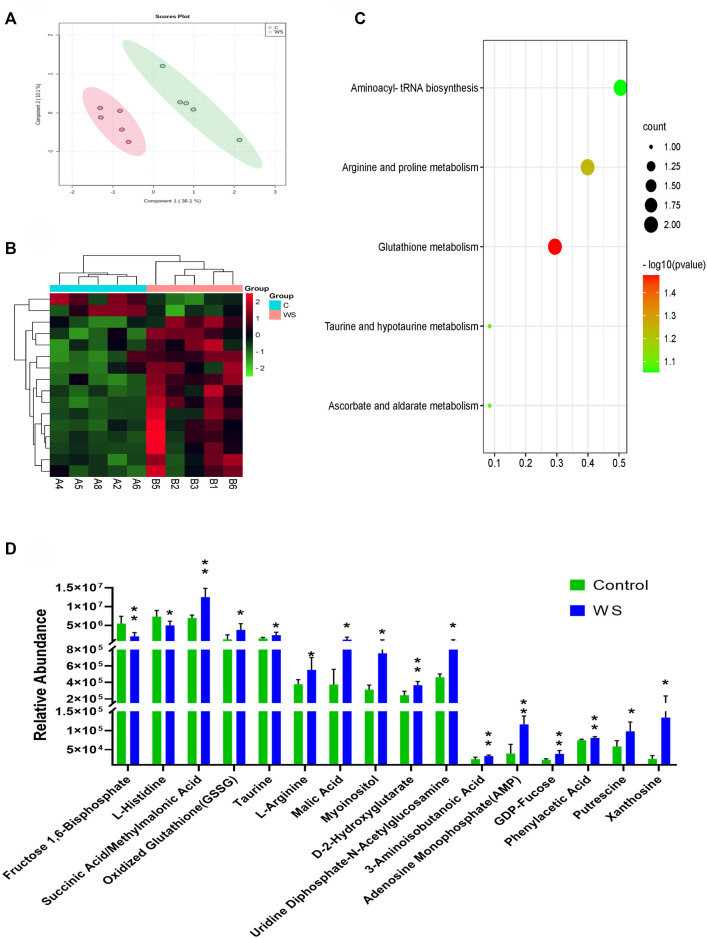
Targeted metabolomic analysis of moderate WS and controls in pure line B. **(A)** PLS-DA score plots of individual metabolites moderate WS and controls. **(B)** Heat map and **(D)** changes in remarkably different metabolites in the breast muscle affected by WS. **(C)** Overview of pathway enriched with significant metabolites in the breast muscle (*p < 0.05, **p < 0.01).

A total of 98 compounds in breast fillets in pure line B were determined (Supplementary Table S4). An obvious separation between the WS samples and controls based on PLS-DA is shown in Figure 3A. There were 16 hydrophilic metabolites significantly changed between the WS group and control. The significant metabolites are displayed in heat map analysis (*p* < 0.05; Figure 3B). As shown in Figure 3D, 14 metabolites, including adenosine monophosphate (AMP)/2′-deoxyguanosine 5′-monophosphate (dGMP), GDP-fucose, xanthosine, L-arginine, and oxidized glutathione, increased in the WS group. Only fructose 1,6-bisphosphate and L-histidine significantly decreased in WS. One pathway, glutathione metabolism, was enriched (*p*-value <0.05) using the 16 hydrophilic metabolites (Figure 3C).

## 4 Discussion

Flavor composed of taste and aroma is an important sensory attribute in the overall acceptance of meat (Wang et al., 2019). Aroma is a consequence of various VOCs that are produced by the complex biochemical changes of the inherent composition of the meat (Shahidi et al., 1986; Khan et al., 2015), such as lipid oxidation, Strecker, Maillard reactions and thiamine degradation (Resconi et al., 2013). However, taste is due to non-volatile water-soluble compounds closely related to water-soluble precursors including carbohydrate compounds, peptides, nucleotides, free amino acids and other nitrogenous components (Mottram and Edwards, 1983). In short, the formation of specific meat flavors can be achieved through different reactions among amino acids of meat hydrolysate and cysteine or methionine and glucose (Jayasena et al., 2013). Therefore, any alteration of metabolite and lipid composition may cause changes in flavor.

The E-nose can analyze food objectively by the instrument of “smell” (Shi et al., 2018). E-nose was able to detect changes in chicken meat owing to storage time and temperature according to Boothe and Arnold (2002). E-noses can also be used to distinguish the flavor of different Chinese local high-quality chicken meat (Jin et al., 2021). In this study, we found that moderate WS and control AA broiler samples could be distinguished by an E-nose. The volatile compounds were further detected by GC-MS in detail. Moderate WS breast contained about 70% of the o-xylene, benzene, 1,3-dimethyl and 2-heptanone and 6-methyl found in the control group. Acetic acid and ethyl ester in moderate WS decreased significantly to 48% of normal levels. The four volatile compounds did not belong to the major volatile compounds found in chicken; 2-methyl-3-furanthiol is reported as the most important compound in chicken flavor and aldehyde is the major one in chicken meat (Jin et al., 2021). In this study, there was no significant difference in aldehyde content between WS and normal groups. This indicates that the major content of WS volatile compounds did not change in moderate WS meat.

As for the 150 pure line chickens, the muscle TG remarkably increased in the WS group. Previous studies also evidenced that the WS samples were increased lipidosis (Kuttappan et al., 2013; Baldi et al., 2018), and moderate WS samples exhibited higher lipid content compared with control samples (Carvalho et al., 2021). Based on the results obtained from lipidomic analysis of pure line broiler chickens, 83.3% of TGs increased, 37.7% of PCs and 28.6% of PEs decreased in the WS meat. Some studies have demonstrated that phospholipids, not TGs, contributed to the sensory aroma in beef (Mottram and Edwards, 1983). Our findings suggested a decreasing pattern in phospholipid flavor precursors in the moderate WS breast fillets. Phospholipids, mainly composed of PCs and PEs and the lipolysis PE, were the main contributor to the increase of free fatty acids during the formation of meat flavor of Nanjing dry-cured duck (Xu et al., 2008). In this study, there are 38 PEs (28.6%) that were decreased significantly and this may be related to the limited changing of VOCs found in the WS tested. The samples for VOCs and lipidomic measurements were from different chicken flocks, which could also influence the consistency of the results. The specific relationship between lipid changes and volatile flavor substances needs further study.

In food, umami taste plays a important role in consumer satisfaction and it is derived from several non-volatile compounds with regard to amino acids, predominantly glutamate and aspartic acid and 5′-nucleotides such as AMP, inosine-5′-monophosphate (IMP) and GMP (Chen and Zhang, 2007). The water-soluble flavor precursors make a important contribution to the umami taste (Mottram, 1998). IMP contributes to the taste of chicken meat (Fujimura et al., 1996; Vani et al., 2006) and ribose, ribose-5-phosphate, glucose and glucose-6-phosphate are the main carbohydrates with flavor-forming potential (Meinert et al., 2009). In this study, AMP/GMP, xanthosine and GDP-fucose increased significantly and fructose 1,6-bisphosphate decreased significantly in the WS meat. By One-dimensional ^1^H-NMR, previous research showed that malonate, taurine, threonine, and arginine were important metabolites for distinguishing normal breast from severe WS (Cônsolo et al., 2020). The inconsistency results showed in two studies may be related to the different analysis methods and the sample conditions. A certain lysine to arginine ratio decreases cooking loss and increases the water holding capacity of pork sausage (Zheng et al., 2017). Histidine is the second largest contribution to taste activity value (TAV) in local chicken breast muscles (Zhao et al., 2020). The increased arginine and decreased histidine of WS found in the current study may also affect the final taste of the breast meat. Thus, changes in water-soluble metabolites may affect the flavoring and substance composition of moderate WS meat. There are still some limitations in our study that volatile compounds and lipids were tested in different sample collections. More studies are required to further explore the flavor precursor alterations related to chicken white striping symptom.

## 5 Conclusion

GC-MS results showed that four volatile compounds, o-xylene, benzene, 1,3-dimethyl, 2-heptanone, 6-methyl and acetic acid, ethyl ester, were found to be decreased in WS meat. Lipidomic analysis showed that the WS meat featured increased neutral lipid, decreased phospholipid molecules. Targeted metabolomic analysis indicated that 16 hydrophilic metabolites were altered in the WS meat. In the WS group, water-soluble flavor precursors, such as L-arginine, AMP/dGMP, xanthosine and GDP-fucose increased significantly but fructose 1,6-bisphosphate and L-histidine significantly decreased. These results provided a systematic evaluation of the flavor precursors in the WS meat of broiler chickens.

## Data Availability

The original contributions presented in the study are included in the article/Supplementary Material, further inquiries can be directed to the corresponding authors.
